# Virtual Reality as a Promising Strategy in the Assessment and Treatment of Bulimia Nervosa and Binge Eating Disorder: A Systematic Review

**DOI:** 10.3390/bs7030043

**Published:** 2017-07-09

**Authors:** Marcele Regine de Carvalho, Thiago Rodrigues de Santana Dias, Monica Duchesne, Antonio Egidio Nardi, Jose Carlos Appolinario

**Affiliations:** 1 Institute of Psychology, Federal University of Rio de Janeiro, Avenida Pasteur, 250, Rio de Janeiro 22290-902, Brazil; 2 LABPR, Institute of Psychiatry, Federal University of Rio de Janeiro, Rio de Janeiro 22290-140, Brazil; antonioenardi@gmail.com; 3 Eating Disorders and Obesity Group, Institute of Psychiatry, Federal University of Rio de Janeiro, Rio de Janeiro 22290-140, Brazil; thiagorjdias.insight@gmail.com (T.R.d.S.D.); monica@duchesne.com.br (M.D.); jotappo@gmail.com (J.C.A.)

**Keywords:** virtual reality, systematic review, binge–purging eating disorders, bulimia nervosa, binge eating disorder

## Abstract

Several lines of evidence suggest that Virtual Reality (VR) has a potential utility in eating disorders. The objective of this study is to review the literature on the use of VR in bulimia nervosa (BN) and binge eating disorder (BED). Using PRISMA (Preferred Reporting Items for Systematic Reviews and Meta-Analyses) statement for reporting systematic reviews, we performed a PubMed, Web of Knowledge and SCOPUS search to identify studies employing VR in the assessment and treatment of BN and BED. The following search terms were used: “virtual reality”, “eating disorders”, “binge eating”, and “bulimia nervosa”. From the 420 articles identified, 19 were selected, nine investigated VR in assessment and 10 were treatment studies (one case-report, two non-controlled and six randomized controlled trials). The studies using VR in BN and BED are at an early stage. However, considering the available evidence, the use of VR in the assessment of those conditions showed some promise in identifying: (1) how those patients experienced their body image; and (2) environments or specific kinds of foods that may trigger binge–purging cycle. Some studies using VR-based environments associated to cognitive behavioral techniques showed their potential utility in improving motivation for change, self-esteem, body image disturbances and in reducing binge eating and purging behavior.

## 1. Introduction

Eating disorders (EDs) are widespread, disabling and often chronic mental disorders [1,2,3]. They are categorized, according to DSM-5 [4], as a group of conditions characterized by a persistent disturbance of eating or eating related behaviors resulting in altered consumption or absorption of food that impairs physical health and psychosocial functioning. The EDs diagnostic subgroup includes major discrete clinical entities such as anorexia nervosa (AN), bulimia nervosa (BN), binge eating disorder (BED) and their subthreshold syndromes. In general, patients with AN could be described by low body weight, food restriction, fear of becoming fat and an overvaluation of their weight/shape (body image (BI) concerns); in those with BN, the core symptomatology are the presence of recurrent binge eating (BE) episodes followed by inappropriate compensatory behaviors (self-induced vomits, fasting, etc.) to prevent weight gain and a self-evaluation unduly influenced by body shape/weight; and, subjects with BED also have recurrent BE but did not engage in compensatory behaviors as those seen in BN. 

Based on several recent clinical and neurobiological findings a spectrum classification model has been proposed which distinguishes between eating disordered patients who restrict food intake without binging and those who binge and purge [5,6]. This distinction between restrictors and binge–purgers is thought to correspond to important phenomenological and etiological differences, including those related to predominant personality features, family-interaction styles, neurobiological abnormalities, and patterns of genetic transmission. Such differences imply that the restrictors versus binge–purgers distinction may delineate more homogenous subgroups of EDs in a more meaningful way regarding treatment [7]. 

Thus, BN and BED are classified as a binge–purging type of ED [6] and both are characterized by a unique phenomenon called BE, a pattern of disordered eating which consists of episodes of unusually large consumption of food associated with feelings of loss of control [4]. Moreover, even though body dissatisfaction was not included as a BED diagnostic criterion, several authors reported high levels of this symptomatology in this population [8]. Along with this core eating psychopathology, patients in binge–purging spectrum disorders usually displayed additional general psychopathology such as depression, anxiety, low self-esteem, and personality traits such as impulsivity. 

Although, currently, the pathophysiology of BN and BED is not clearly understood, several hypotheses based on these emotional aspects have been proposed for the development and maintenance of these disorders [9,10] and may represent a potential path for the development of new interventions. In this perspective, BI disturbances/dissatisfaction may potentially lead to feelings of negative affect and attempts to reduce caloric intake, and in turn, negative affect and dietary restraint lead to BE [9]. More recently, certain personality traits as impulsivity have been implicated in triggering eating disordered eating. Impulsivity might be related to deficits in inhibitory control, i.e., the capacity to delay a response, to suppress inappropriate responses and to ignore distractions [11,12]. A growing body of evidence suggests that impulsivity and reduced inhibitory control are associated with overeating and binge-eating behavior [13,14].

BN and BED have a complex, multifactorial etiology, involving the interactions between genes and environment, with an interplay of biological, psychological and sociocultural factors. Although psychotherapeutic approaches (such as cognitive-behavior therapy (CBT)) are the most effective and recommended first-line treatment for those conditions, in general, currently available treatments have been shown only moderately effective. Thus, there is an urge to improve the understanding of EDs etiology and to develop a more efficacious and personalized forms of interventions [15]. 

Virtual reality (VR) is an emerging technology with a variety of uses ranging from clinical research to the assessment and treatment of several medical and psychological conditions [16]. A comprehensive definition for VR is “an advanced form of human–computer interface that allows the user to interact with and become immersed in a computer-generated environment in a naturalistic fashion” [17]. An important aspect of VR is that it induces a kind of “sense of presence”, i.e., a feeling of “being there” in the middle of the virtual environment. This sensation of presence produces a setting where the subject can stay and live a specific experience, evoking emotional responses and a sense of self-reflectiveness [18]. Summarizing, VR reduces the gap between reality and imagination and have a potential utility in behavioral health for the assessment and treatment of mental disorders, because it allows the individual to face anxiogenic situations in a more controlled environment [19]. 

Initially designed for treatment of phobias, the use of VR in behavioral disorders has expanded for other mental health conditions [20]. Freeman and colleagues systematically reviewed the studies of VR in mental disorders and found that the main conditions investigated were anxiety disorders, schizophrenia, substance related disorders and EDs [16]. The authors pointed out that VR environments can be used to elicit psychiatric symptoms, manipulation of VR can inform the understanding of disorders, and simpler psychological treatments can be successfully administered in VR format. Another systematic review focusing specifically on the potential use of VR in interventions for mental disorders [21] demonstrated that, overall, VR has been shown superior to treatment as usual or waiting lists and with similar efficacy as conventional CBT or in vivo exposure. However, it is important to mention that the effectiveness of VR-based interventions varied across diagnostic groups and the studies showed several methodological flaws. Both reviews [16,21] commented that EDs is a promising area for utilization of VR.

In general, VR is used in EDs studies exploring two main aspects of the EDs syndrome: BI distortions and uncontrolled eating. For the first aspect, it usually enables three-dimensional figures of the patient’s body to be presented, helping him/her to reach an awareness of BI distortion and then providing the possibility to face and modify distortions, which results in a more realistic view of the body and a potential decrease in BI dissatisfaction. For the second topic, VR provides scenarios that simulate real-life situations and to encounter food cues known to trigger his/her disordered eating behavior. Riva and colleagues [20] pioneered the studies on the use of VR for the treatment of EDs as part of a collaborative network of the European VREPAR PROJECT (VR Environments for the Psychoneurophysiological Assessment and Rehabilitation Project). They developed the VEBIM (Virtual Environment for BI Modification), composed of five VR environments for the treatment of BI disturbance. 

Ferrez-Garcia and colleagues [22] in 2013 summarized the available information regarding the use of VR for the treatment of EDs and obesity. From the 17 studies included in this systematic review, 11 investigated VR-based interventions specifically in patients with EDs. In terms of the diagnostic rubrics of the samples and designs of the studies, there were four case reports describing the effects of VR in AN subjects, two non-controlled and two controlled trials with patients with BED; two controlled studies with a mixed sample of AN and BN individuals; and one non-controlled study in an eating disorder not elsewhere specified (EDNOS) sample. In the same year, reviewing exposure therapy in the field of EDs, Koskina and colleagues [23] analyzed the existing evidences of food and BI exposure using VR environments. Taking together, these reviews considered that treatment studies with VR in EDs are emerging. Although the authors are generally supportive of the potential usefulness of VR in EDs they highlighted several methodological limitations of the current studies that need to be overcame in the future research. 

To update the current knowledge on the use of VR-based environments in a more homogenous subgroup of binge–purging EDs we performed this systematic review in order to summarize the current evidences of VR in the assessment and treatment of BN and BED.

## 2. Methods

Using the PRISMA statement for reporting systematic reviews, we performed a PubMed, Web of Knowledge and SCOPUS search to identify studies using VR in the assessment and treatment of BN, BED and their subthreshold syndromes published until June 2017. Two independent reviewers (M.R.C. and T.R.S.D.) performed study selection procedures and data extraction. The decisions on inclusion or exclusion of the reviewers were recorded. The results from the data abstraction were compared after completing the review of the articles. Any discrepancies or disagreements were resolved by consensus, with attention to the previously defined selection criteria. A third reviewer would decide upon inclusion or exclusion in the case of a persistent disagreement between the reviewers. We searched for original clinical studies (controlled and uncontrolled) investigating the utility of VR environments in samples that includes patients with the diagnosis of BN and BED. Thus, if the sample of the study along with BN and BED also included patients with other ED rubrics (such as AN or EDNOS) the paper was incorporated. We included also case-reports. The bibliography of review articles was used to find articles not retrieved by the electronic search. The following combination of search terms were used: “virtual reality AND eating disorders”, “virtual reality AND binge eating”, and “virtual reality AND bulimia nervosa”. The selection of papers suitable for this review was restricted to articles published in peer-reviewed journals. Where a title or abstract seemed to describe a study eligible for inclusion, the full article was obtained and examined to assess relevance based on the inclusion criteria. Subsequently, the articles were divided according to the study design, data extracted and organized in tables.

## 3. Results

**Figure 1:**
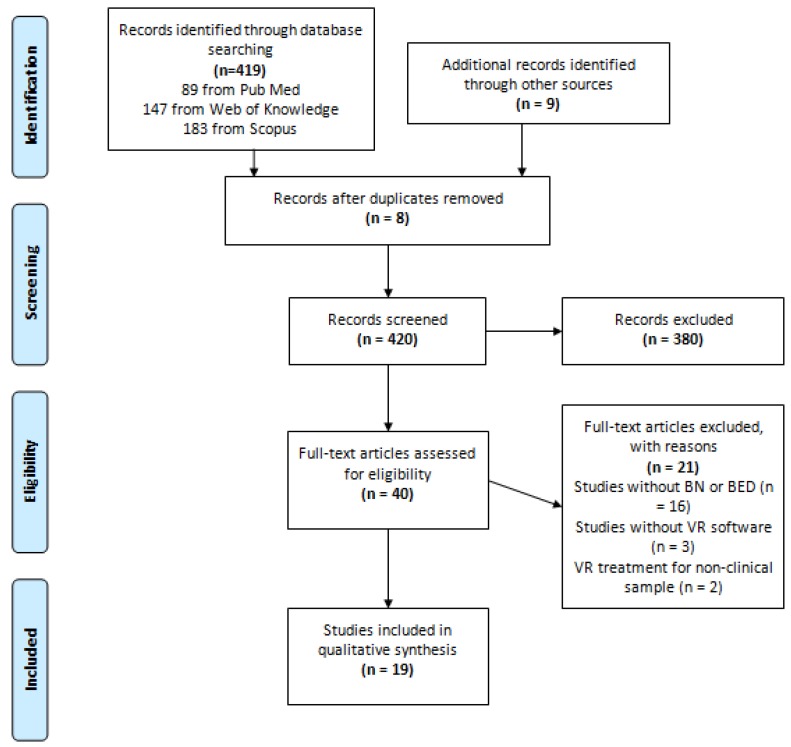
PRISMA Flow Diagram.

The initial electronic search identified 420 studies using VR-based environments in patients with BN and BED. Additionally, nine studies were identified by hand searching the references of the original and review articles retrieved. After a careful process of appraisal, 19 studies met the inclusion criteria and were selected and included in this review (see Figure 1). From these 19 studies using VR in BN and BED, nine were classified as *assessment studies* [24,25,26,27,28,29,30,31,32]—which includes studies of symptom assessment, identification of symptom markers, tests of putative causal factors and other studies investigating different aspects of the conditions—(see Table 1) and 10 as *treatment studies* [33,34,35,36,37,38,39,40,41,42]—reports and studies with includes VR-based environments as a therapeutic intervention—(see Table 2).

In general, an important aspect of those studies is that they have been conducted by basically three group of researchers using different VR-systems. Through six VR environments, which included exposure to food and social situations, assessment studies were mainly conducted by Gutiérrez-Maldonado et al. [24,25,26,28,30,31]; VEBIM developed by Riva et al. [38,43] with several updates such as VEBIM 2 or VREDIM (Virtual Reality for Eating Disorders Modification); and Virtual & Body developed by Perpiñá et al. [33], The latter two software packages were used in assessment and treatment studies. BI disturbance was the main target of the VR component in these interventions, and body dissatisfaction was the primary outcome variable assessed. Other outcomes often included were depression, self-esteem, assertiveness, and other symptoms related to ED. Considering the assessment studies from the nine studies included in this review, only two included a homogeneous sample of BN and BED individuals, while the others comprised mixed samples of restrictive and binge–purging type of EDs. Conversely, the treatment studies were less heterogeneous and, apparently, only two studies included mixed samples.

In the following paragraphs, we revised the selected studies in more detail.

### 3.1. VR in the Assessment of BN and BED

Among the umbrella of the assessments studies we found investigations trying to characterize the differential responses of EDs patient’s symptomatology to BI and food related VR settings and to validate that these apparatuses really provide a real sense of presence. In this sense, Gutiérrez-Maldonado et al. [24] and Ferrer-García et al. [25] showed that ED patients presented higher anxiety when exposed to high-calorie food situations and to locations such as a swimming-pool when compared to a neutral environment. In another study, Gutiérrez-Maldonado et al. [26] reported that ED patients showed more BI distortions and body dissatisfaction facing high-calorie food environments when compared with in low calorie food environments. In relation to the sense of presence, a group of studies [27,28,29] have demonstrated that, while exposed to the virtual environments, ED patients experienced satisfactory sense of presence. Gorini et al. [27] confirmed this impression and reported that the higher sense of presence experienced in VR settings by a group of patients with AN and BN was associated with higher levels of anxiety. 

More recently, assessment studies have investigated virtual food environments (food-cues) in terms of triggering, maintaining and/or relapsing mechanisms associated to BN and BED. They showed that exposure to virtual food could induce similar reactions as exposure to real food, and that VR environments relevant to ED were able to elicit emotional, cognitive and behavioral responses in these patients. Perpiñá et al. [29] exposed ED patients to a virtual kitchen where they could prepare and eat food. Before eating, patients displayed moderate to high scores of urge to eat, fear, and avoidance; and low desire to eat. After eating, they reported feelings of putting on weight, urge to eat and being upset; and some behavioral aspects such as pressure to exercise as a compensatory measure, to continue eating, and to purge. Gorini et al. [27] verified that virtual food was as effective as real food, and more effective than photographs, in producing psychological and physiological responses in ED patients. Ferrer-García et al. [30] and Pla-Sanjuanelo et al. [31] used a software with four VR scenarios that contained foods chosen by the participants as the ones that produced the highest level of craving, and verified that VR was useful for inducing food craving in BN and BED patients. Participants with higher levels of trait and state-craving showed a greater desire to eat during exposure to virtual foods [31]. 

Ferrer-García et al. [30] and Ferrer-García and Gutiérrez-Maldonado [28] reported that high-calorie food settings and certain social situations produced the highest levels of subjective discomfort. In the study of Perpiñá et al. [29], patients showed a high reality judgment and sense of presence and scored higher on emotional involvement and attention when compared to controls. Perpiñá and Roncero [32] also reported that the highest scores on emotional involvement, attention, reality judgment and presence were obtained by the ED group. They also remark that the level of emotional reactivity to VR proportionally increased the sense of presence in the virtual environment. In contrast with those findings, Ferrer-García and Gutiérrez-Maldonado [28] reported a low sense of presence experienced in ED patients. However, considering those patients that reported greater sense of presence they displayed a higher level of subjective discomfort in food related situations.

### 3.2. VR in the Treatment of BN and BED

#### 3.2.1. Case Report

Roncero and Perpiñá [41] described a case of a 17-year-old woman with BN and assessed the effect of VR as a therapeutic tool to normalize eating patterns as part of a CBT treatment. The patient was treated for seven weeks using VR sessions and Fairburn’s CBTC protocol [44] for BN. The VR software consisted of a kitchen with two areas that contained elements to cook, drink and eat. The patient could access the foods freely or block them if desired. She could also perform alternative behaviors in the virtual environment (for example, making a phone call). At the end of the treatment, the patient showed a reduction in BE, purges and food avoidance. In addition, after VR exposure, the patient showed an overall improvement, such as a decrease in drive for thinness, bulimia measures and in general psychopathology. The authors concluded that VR was an effective complement to CBT treatment in this bulimic patient. 

#### 3.2.2. Non-Controlled Clinical Trials

Riva et al. [35] conduct two studies with female participants, one of them with an obese group and other with BED patients, to evaluate a VR-based treatment to be used in the assessment and treatment of those patients. VEBIM 2 software was used, which includes cognitivebehavioral and visualmotorial methods. VR first session aimed to assess stimulus that elicit abnormal eating behavior, focusing on patient’s concerns about food, eating, shape and weight. The other four sessions intended to assess and modify the symptoms of anxiety related to food exposure and the patient’s body experience. For these purposes, different zones are used, such as sitting-room, dining-room, kitchen, bedroom and working environments. In both studies, patients who ate 1200 kcal per day, besides VR intervention, also participated in bi-weekly psycho-nutritional groups held by dietitians. In both groups, results showed a reduced level of body dissatisfaction, an improvement on social activities and a reduced use of disguising clothes. BI change was associated to a reduction in problematic eating. The authors concluded that the obtained data were promising, but controlled and follow-up studies are needed to test this VR approach. Riva et al. [34], in another paper, reported again both clinical trials of the previous study Riva et al. [35] with obese and BED patients and extended the sample conducting another non-controlled clinical trial, with the same objective and design, with EDNOS patients. The results pointed out to a reduction on body dissatisfaction, but it was slighter than the observed in other samples. The patients improved on social activities and reduced disguising clothes’ use. Again, authors concluded that VR-based treatment could be useful to BI modification in obesity, BED and EDNOS; however, more studies are needed. 

#### 3.2.3. Randomized Controlled Trials

Perpiñá et al. [33] conduct a randomized controlled trial with AN and BN female patients to measure the effectiveness of the treatment of BI through VR. Patients were assigned to two conditions: cognitive-behavioral treatment plus VR exposure or cognitive-behavioral treatment plus relaxation. The VR software consisted of six areas where the patients could experience discrepancies about weight and BI: an accommodation zone; a food area with a virtual balance (to discuss the discrepancies about subjective, desired and healthy weight; and to address overestimation of weight after eating); a room with posters showing different body shapes (to help the patient understand that weight is a relative concept); a room with mirrors (to deal with the representation of BI); a doorframe with strips (to deal with the estimated body size); and another zone to contrast body areas (to contrast subjective body shape, desired body shape and body shape that the patient thought that a significant person would have of her). The patients moved through the VR settings according to their progress. CBT plus VR group showed significantly improvement in general psychopathology, ED related symptoms and BI aspects (less weight fear, higher satisfaction with their bodies in social contexts, less thoughts and negative attitudes about their bodies, and less fear of achieving a healthy weight) when compared to CBT plus relaxation group. The results showed that greater improvement was achieved through the addition of VR to the treatment of BI.

To evaluate the efficacy of a VR-based multidimensional approach in the treatment of BI disturbances, Riva et al. [36] conduct a randomized controlled trial with female BED patients involved in a residential weight control treatment, which included a low-calorie diet (1200 Kcal/day) and physical training. Patients were assigned to two conditions: VR-based treatment and a psycho-nutritional intervention. VREDIM software was used, an enhanced version of the VEBIM environment, that includes cognitive-behavioral and visual-motorial methods. The software consists of different zones: room with balance, drawing room, kitchen, dining room, bathroom, office, beach, supermarket etc. Initially the focuses of the intervention were patient’s concerns about food, eating, shape, and weight. Then, patients had to answer the “miracle question”; that is, they were asked to imagine what life would be like without their complaint. They built their solution, which was used to guide the therapeutic process. Next, the focuses are to manage anxiety symptoms related to food exposure and body experience. The psycho-nutritional group was also based on a cognitive-behavior approach, applied with the purpose of teaching methods for stress management, problem-solving and eating behavior. Results for VR treatment showed an overall psychological status improvement, reduced level of body dissatisfaction and anxiety, increased self-efficacy and motivation for change, reduced concern about social judgment and reduced overeating. The control group presented anxiety reduction on the Assertion Inventory (AI), but it was not confirmed by the State-Trait Anxiety Inventory (STAI) score. VREDIM was more effective than psycho-nutritional group on body satisfaction improvement, overeating and anxiety level reduction. Patients of both groups had remission of BE episodes at the end of the treatment. VR-based therapy improved BI (and its associated behaviors) treatment of patients involved in a weight control program with physical training. 

Riva et al. [37] in a randomized controlled trail with female BED patients compared the efficacy of three interventions (ECT: experiential cognitive therapy, CBT, and NG: nutritional group) with a waiting list, and also observed the outcomes over a six-month follow-up period. ECT used VREDIM software and included nutritional intervention and physical training. CBT was based on Fairburn’s [45,46] protocol and included group sessions to improve assertiveness and motivation to change, as well as nutritional intervention and physical training. Nutritional group included a low-calorie diet (1200 Kcal/day), prescribed by dieticians, and physical training. Results showed that ECT helped with the decrease of anxiety and depression levels and improved assertive behavior. CBT decreased patients’ depression level and NG decreased anxiety level. Patients of all interventions groups improved on self-esteem, eating control, eating self-efficacy and weight loss. They also reduced binge episodes. ECT was more effective than CBT in improving overall psychological state, BI, body satisfaction and resistance to social pressure. At a six-month follow-up, ECT improved body satisfaction and self-esteem and reduced the frequency of binge episodes of BED patients when compared to CBT and NG. VR-based therapy improved BED patient’s treatment when compared to other conditions.

Riva et al. [38] reported a clinical trial with female patients with obesity, BED, BN and EDNOS comparing ECT, CBT, nutritional group and a control group. ECT used VREDIM software and also included nutritional intervention and physical training. CBT was delivered with a nutritional intervention and physical training. Nutritional group included psychological support and physical training. For ED patients, at post-treatment, ECT decreased anxiety and depression levels and improved assertive behavior. CBT decreased depression level and NG intervention decreased anxiety. Otherwise, the waiting list group had increased anxiety level and weight gain. All interventions groups improved patients’ self-esteem, eating control, eating self-efficacy and weight loss. When all groups were compared, ECT was the best intervention for eating control improvement. ECT and CBT were better on improving body satisfaction and body perception. ECT was better than CBT on BI and self-efficacy improvement. Besides, for obese patients, at post-treatment, ECT and CBT decreased depression levels and improved self-esteem and assertive behavior. All interventions helped to decrease patients’ anxiety and improved eating control and eating self-efficacy. When compared, all interventions groups had body satisfaction and body perception improvement. ECT was the best intervention for eating control improvement. ECT and CBT were better in improving motivation to change. ECT was better than CBT in BI improvement. The authors concluded that ECT was more effective than the others approach in the treatment of obese, BED, BN and EDNOS patients.

Perpiñá et al. [42] examined the evolution of the results of a previous study carried out by her research group [33] over six-month and one-year follow-up periods. The group that received CBT plus relaxation in the first study received VR sessions after that treatment, so in this follow-up study the entire sample had completed the treatment in the VR condition. The results at post-treatment were maintained at one-year follow-up, and for some measures such as appearance-related schemas and ED related components the improvement continued along the follow-up period. General psychopathology improved from pre-treatment to one-year follow-up, and The Brief Symptom Inventory (BSI) score was always below the pre-treatment level, but it rose between 6 and 12 months. VR was useful in the treatment of BI and can enhance the efficacy of the standard CBT.

To compare CBT treatment for ED with a CBT protocol focused on BI treatment plus VR, Marco et al. [39] enrolled AN, BN and EDNOS female patients to two randomized groups over a one-year follow-up study. CBT for AN followed Garner et al. [47] protocol, CBT for BN was based on Wilson et al. [48], and CBT for EDNOS followed one of these two protocols, according to each patient needs. CBT for BI treatment was adapted from Butters and Cash [49] and Perpiñá et al. [50]. VR software consisted of five situations: virtual scale and a kitchen, a room with posters, a room with mirrors, a doorframe with strips and a zone to contrast body areas. The purposes of the exposure to each environment were described above Perpiñá et al. [33] VR treatment group showed more BI improvement than CBT and greater improvement in the behavior clinical measures. At post-treatment, VR group improved on body attitudes, frequency of negative automatic thoughts on BI, body satisfaction, discomfort caused by body-related situations and BN symptoms (measured by Bulimic Investigatory Test; BITE). These results were maintained or continued to improve (body attitudes, frequency of negative automatic thoughts on BI) at one-year follow-up. All participants improved in the ED measures and it was maintained at follow-up. The authors analyzed the clinical effectiveness of both treatment conditions. Patients of VR group showed clinically significant improvement on post-treatment and follow-up and all BI and ED scores were similar to those of the healthy population. Their Eating Attitudes Test (EAT) scores were better than the healthy population. In addition, BITE normalization was achieved at follow-up. Otherwise, patients of CBT group did not show clinically significant improvement at post-treatment or follow-up. In conclusion, CBT focused on BI treatment plus VR improved CBT standard treatment for ED.

Cesa et al. [40] in a randomized controlled trial with a one-year follow-up, tested the efficacy of CBT including a VR protocol (ECT) in morbidly obese patients with BED compared with CBT alone and an inpatient multimodal treatment (IP), that included psycho-nutritional groups, a low-calorie diet (1200 Kcal/day), and physical training. In fact, both other groups also received the IP. CBT protocol was based on Fairburn et al. [44] and Ricca et al. [51]. NeuroVR software was used, which includes 14 virtual environments that present situations related to maintaining and relapse mechanisms (Home, Supermarket, Pub, Restaurant, Swimming Pool, Beach, Gymnasium) and BI comparison areas. All patients had a reduction in weight, BE episodes (decreased to zero) and improved body satisfaction. Only patients of ECT improved on BI concerns. ECT and CBT were more effective than IP alone in preventing weight regain at follow-up, and only ECT was effective in patients’ further weight loss. At follow-up BE episodes were reported in all groups, but ECT and CBT groups were successful at maintaining them at a low rate. VR based therapy showed some advances when compared to other groups at post and follow-up treatment. 

## 4. Discussion

This systematic review on the use of VR based environments in the assessment and treatment of BN and BED expanded the information provided by previous reviews [16,21,52] by focusing in a more homogenous group of binge–purging EDs and adding more recent investigations in the area [30,31,32,41]. From the 18 studies selected, nine were characterized as assessment studies because they investigated different aspects of these syndromes using VR-related settings, and nine were considered treatment studies by exploring the usefulness of VR as a potential therapeutic strategy in BN and BED. Based on the studies selected, the use of VR in EDs is an evolving area and, although in an early stage, the results of current studies are promising. First, the overall findings support the idea that patients with BN and BED immersed in VR environments felt a sense of presence, improving the ecological validity of this procedure in this group of individuals. Secondly, considering the available evidences the use of VR in the assessment of BN and BED showed some promise to identify: (1) how those patients experienced their BI; and (2) and environments or specific kind of foods that may trigger binge–purging cycle. Finally, the studies using VR technologies associated to cognitive behavioral procedures demonstrate their potential utility in improving eating related (binge-eating, urge to eat, etc.), general psychopathology and other aspects of these binge–purging conditions. Studies showed that VR assessment tools provide novelty features when compared to available psychometric tests. Through VR systems it is possible to manipulate the size and shape of body areas and to compare the visualization of the differences between the actual, perceived and desired body weight or shape of ED patients [27]. Psychometric instruments can collect information through patients reporting, but VR can turn these reports into concrete perceptions. Other advantage is that the perception of presence in the virtual environments can elicit compulsive behaviors in a way like reality, and this is a benefit when studying the triggers of BE and the response to them. 

VR treatment can present some advantages over reality, but its objective is not to substitute real experiences. Virtual exposure has been considered an alternative for imaginary exposure and an intermediate step for in vivo exposure. If experienced anxiety is too high, receiving a virtual exposure treatment can increase patients’ possibilities of accepting an in vivo exposure program in the future [53]. In addition, exposures could be detailed customized by the therapist in the VR exposure condition, which can help patients to cope with stimulus or complex environments in a more feasible and controlled way. The possibility of monitoring the responses of the patient also offers an advantage over real exposure [54,55]. It was already argued that patients may progress more rapidly in their VR exposure hierarchy due to a perception of increased control and safety [55]. Besides these advantages, these VR based apparatus are accessible and have a good cost-effectiveness ratio. Just to give an example, the VR systems used in BN BED studies used affordable equipment such as a basic computer hardware (1 GB RAM, 256 Mb graphic card, and 17-in) sold for about USD 500,00 (unit); and VR glasses, with an approximate value of USD 700,00 (unit). In addition, the Neuro VR [40] software is available for free download at http://www.neurovr2.org/. 

The rationale of the use of VR exposure is that it is supposed to work according emotion-processing model. The confrontation with a threatening stimuli (which elicits the fearful responses) activates the fear network. The processes of habituation and extinction aid modification of the fear structure, making its meaning less threatening, as new and incompatible information is added into the emotional network [20]. Thus, VR exposure can reduce eating-related anxiety during and after exposure to virtual foods and environments, helping to disrupt the reconsolidation of adverse food/situations related memories [23,31]. For the exposure therapy, the hierarchy is created in the software, and patients are first exposed to stimuli that elicit the lowest levels of craving until they can reach the ones that elicit the highest levels. During the exposure, the level of craving is assessed, and the patient must stay in the situation until the levels of craving and anxiety diminish sufficiently. Then, the patient can cope with the following stimuli in the hierarchy. 

Another reason for the use of VR is related to the Allocentric Lock Theory, which suggests that ED may be associated with impairment in the ability to update a stored negative allocentric (offline) representation of one’s body with real-time (online/egocentric) perception-driven inputs [56,57]. The addition of VR sensory training to unlock the body memory (body image rescripting protocol) by increasing the contribution of new egocentric/internal somatosensory information directly related to the existing allocentric memory was able to improve the efficacy of CBT at one-year follow-up, supporting its rationale [40,58]. In addition, Riva et al. [54] emphasizes the role of VR in transforming external and internal experience. External experience can be modified by focusing on the personal efficacy and self-reflectiveness, and both can be achieved through the sense of presence and emotional engagement that the exposure to virtual environments can provide. Besides, inner experience can be modified by structuring, altering, and/or replacing bodily self-consciousness.

It is important to register that, after a first phase of immaturity (published between 1999 and 2004) with studies showing several methodological flows the field has evolved. The more recent studies, in particular the ones published in the last five years follow the guidelines required by high level RCTs. Two different randomized, controlled trials [39,40] have shown at one-year follow-up that VR had a higher efficacy than the gold standard in the field, i.e., cognitive behavioral therapy (CBT). 

In line with the theoretical framework mentioned previously, the rationale behind the VR systems currently used in the ED studies incorporated environments to deal with BI distortions [59] and regulation of emotions [25]. More recently, settings offering exposure with response prevention of bingeing have been also included [30]. The novelty of this proposal is the addition of VR to cue-exposure procedures. Food cue-exposure therapy (CET) has been proposed as an effective treatment for binge–purging syndromes, eliminating or significantly reducing binging and purging behaviors [60]. 

Future recommendations included the need for more high-quality RCTs, [61], with robust sample sizes and long-term follow-ups to investigate the effects of VR-based interventions in BN and BED. Forthcoming studies should also compare VR-based interventions in more homogenous groups of BN and BED patients (and not in mixed samples as observed in this review) with waiting lists and active interventions such as psychotherapy and pharmacotherapy. To facilitate comparability of treatments, standardized outcome measures (instruments to assess eating-related and general psychopathology) should be used in line with current available literature on other interventional studies in BN and BED. 

A limitation of this study was the impossibility to report separately the results related to binge–purging EDs. The objective of the review was to exclude studies with AN-only subjects, as this subgroup of restrictive type of eating disorders have a distinctive pathophysiology. However, currently, as there are few studies with BN and BED-only samples, mixed samples were incorporated. Unfortunately, specific information related with binge–purging EDs were not possible to be selected because of the lack of report and analysis of each group of patients separately in the primary studies results. 

## 5. Conclusions

In conclusion, based on the current available data VR-based environments may be considered a promising strategy for the assessment and treatment of BN and BED. Considering the available evidence, the use of VR in the assessment of those conditions showed some promise in identifying: (1) how those patients experienced their body image; and (2) environments or specific kind of foods that may trigger binge–purging cycle. VR-based environments associated to cognitive behavioral techniques showed their potential utility in improving motivation for change, self-esteem, body image disturbances and in reducing binge eating and purging behavior.

## Figures and Tables

**Table 1:** Studies using virtual reality in the assessment of bulimia nervosa and binge eating disorder.

Study	Sample Size (M/F)	Diagnostic	Study Design	Intervention	Software Characteristics	Sessions	Follow-up	Instruments	Outcomes
Gutiérrez-Maldonado et al. (2006) [24]	30 (0/30) AN: 17 (0/17) BN:11 (0/11) EDNOS: 2 (0/2)	AN BN EDNOS	NCAS	Exposure to food and people.	6 VR environments: living-room, kitchen with high calorie food, a kitchen with low-calorie food, a restaurant with high-calorie food, a restaurant with low-calorie food, swimming-pool.	1	-	STAI, CDB, EDI-2, PQ.	Higher state anxiety in the high-calorie food situations and the swimming-pool than in the neutral environment. Higher depressed mood in the high-calorie food situations. Significant differences on the level of state anxiety and depression mood comparing low-calorie and high-calorie food environments; no differences were found between environments with people and those without. AN and BN patients responded with similar levels of emotional intensity to the different situations. VR was useful for eliciting emotional reactions
Ferrer-García et al. (2009) [25]	193 (0/193) ClG: 85 (0/85) (AN: 49, BN:22, EDNOS:14) CG: 108 (0/108)	AN BN EDNOS	CAS	Exposure to food and people.	6 VR environments: neutral, kitchen with high calorie food, a kitchen with low-calorie food, a restaurant with high-calorie food, a restaurant with low-calorie food, swimming-pool.	1	-	EAT, STAI, CDB.	ED patients showed significantly higher levels of anxiety and depressed mood in the high-calorie food environments and the swimming pool than in the neutral room. ED patients’ anxiety increased when other people were present, but in high-calorie environments, their anxiety increased when they were alone. ED patients showed a more depressed mood after eating low-calorie food when other people were present. After eating high-calorie food, they felt more depressed when they were alone. VR was useful for eliciting emotional reactions.
Gutiérrez-Maldonado et al. (2010) [26]	193 (0/193) ClG: 85 (0/85) (AN: 49, BN:22, EDNOS:14) CG: 108 (0/108)	AN BN EDNOS	CAS	Exposure to food and people.	4 VR environments: kitchen with high calorie food, a kitchen with low-calorie food, a restaurant with high-calorie food, a restaurant with low-calorie food.	1	-	EAT, BIAS.	ED patients showed more BI distortion and body dissatisfaction in the high-calorie food environments than in the low calorie food environments. People variable was not significant. BN patients showed greater BI distortion when were alone after eating high-calorie food than after eating low-calorie food. Where the patient was accompanied, BI distortion levels were similar, regardless of the kind of food. AN and EDNOS patients presented higher levels of body distortion after eating high-calorie food than after eating low-calorie food, independently of the presence or absence of people. BI distortion and BI dissatisfaction can be influenced by VR situational factors.
Gorini et al. (2010) [27]	ClG:20 (0/20) (AN:10, BN:10) CG:10 (0/10)	AN, BN (DSM-IV)	CAS	RF PH VR	Small restaurant with a buffet table, 6 foods.	1	-	EDI-2, STAI-S, VAS-A, ITC-SOPI.	Higher level of anxiety for patients compared to control. Patients felt more anxious when exposed to real and virtual food than to the pictures of food. Patients showed higher heart rate and skin conductance compared to control group. Their level of physiological anxiety was higher in the RF and VR condition, than in the PH condition. Higher sense of presence was associated with higher levels of anxiety. Virtual food was as effective as real food, and more effective than photographs, in producing psychological and physiological responses in ED patients.
Ferrer-García and Gutiérrez-Maldonado (2011) [28]	71 (0/71) (AN:49, BN:22)	AN, BN	NCAS	Exposure to food and people.	5 VR environments: neutral room, kitchen with high calorie food, a kitchen with low-calorie food, a restaurant with high-calorie food, a restaurant with low-calorie food.	1	-	EAT-26, PQ.	High-calorie environments and social situations produced the highest levels of subjective discomfort. Patients with severe symptomatology showed a higher subjective discomfort in all environments than with moderate symptoms. Reported sense of presence was low. Patients with high sense of presence showed the highest levels of subjective discomfort in all food situations.
Perpiñá et al. (2013) [29]	ClG:22 (0/22) (AN:11, BN:4, EDNOS:7) CG:37 (0/37)	AN, BN, EDNOS (DSM-IV-TR)	CAS	Exposure to food.	Kitchen with 2 areas: prep area and area with a table and a chair.	1	-	BDI-II, BAI, RS, RJPQ, ITC-SOPI.	Before eating patients showed moderate–high scores on control urge to eat, fear, avoidance; and low desire to eat. After eating, they reported feelings of putting on weight, urge to continue eating, of being upset etc.; and reported wanted actions: to do exercise to “compensate”, to continue eating, to continue with their daily routine, to purge. Patients showed a high reality judgment and sense of presence and scored higher on emotional involvement, attention and negative effects. VR software was clinically meaningful to patients.
Ferrer-García et al. (2015) [30]	ClG: 40 (10/30) (BN:23, BED:17) CG: 78 (9/69)	BN, BED (DSM-5)	CAS	Exposure to food.	4 VR scenarios (kitchen, dining room, bedroom, and bakery/café) + 10 foods (of 30 available foods that elicit craving).	1	-	DEBQ.	BN and BED patients showed higher levels of emotional, external and restrictive eating and food craving than controls. External eating was associated with food craving both in patients and controls. VR was useful for inducing food craving in BN and BED patients.
Pla-Sanjuanelo et al. (2015) [31]	118 (19/99) ClG: 40 (10/30) (BED: 17 BN:23) CG: 78 (9/69)	BED (DSM-5) BN (SCID-I)	CAS	Exposure to Food.	4 VR scenarios + 10 foods (of 30 available foods that elicit craving).	1	-	FCQ-T, FCQ-S.	Participants with higher levels of trait and state-craving showed a greater desire to eat during exposure to virtual foods. State-craving was associated with perceived craving experience in both groups during VR exposure. VR-CET model may be helpful in improving the treatment of BE and BN patients.
Perpiñá and Roncero (2016) [32]	62 (0/62) ED: 20 (0/20) (AN:10, BN:4, EDNOS:6) Obese: 19 (0/19) CG:23 (0/23)	AN, BN, EDNOS (DSM-IV-TR) Obesity	CAS	Exposure to food.	Kitchen with 2 areas: prep area and area with a table and a chair.	1 (30 min.)	-	RJPQ, ITC-SOPI.	ED group had the highest scores on emotional involvement, attention, reality judgment and presence, negative effects. Obese group had the lowest scores on reality judgment and presence, satisfaction, sense of physical space in VE experience. Attribution of reality to the virtual eating was predicted by engagement and belonging to the ED group. The palatability of a virtual food was predicted by attention capturing and belonging to the obese group. The level of emotional reactivity to VR proportionally increased the sense of presence. VR was useful for assessing and measuring ED patients’ responses in a naturalistic setting.

**Note: Participants**—AN: Anorexia Nervosa; BED: Binge Eating Disorder; BN: Bulimia Nervosa; ED: Eating disorder; EDNOS eating disorder not otherwise specified; **Intervention**—CET: Cue Exposure Therapy; PH: Photograph slide show; RF: Real Food view; **Study Design**—AS: Assessment Study; CAS: Controlled Assessment Study; **Instruments**—BAI: Beck Anxiety Inventory; BDI II: Beck Depression Inventory; BIAS: Body Image Assessment Software; CDB: The Barcelona Depression Questionnaire; DEBQ: Dutch Eating Behavior Questionnaire; EAT: Eating Attitudes Test; EDI 2: Eating Disorders Inventory 2; FCQ-T, FCQ-S: State and Trait Food Cravings Questionnaires; ITC-SOPI: Sense of Presence Inventory; PQ: Presence Questionnaire; RJPQ: The Reality Judgment and Presence Questionnaire; RS: Restraint Scale; STAI: Sate-Trait Anxiety Inventory; VAS-A: Visual analogue scale for anxiety.; **Other:** CG: Control Group; ClG: Clinical Group; F: Female; M: Male.

**Table 2:** Studies using virtual reality in the treatment of bulimia nervosa and binge eating disorder.

Study	Sample Size (M/F)	Diagnostic	Study Design	Intervention	Software Characteristics	Sessions	Follow-Up	Instruments	Outcomes
**Treatment**									
Perpiñá et al. (1999) [33]	13 (0/13) AN: 7 (0/7) BN: 6 (0/6)	AN, BN (DSM-IV)	RCCT	SBIT+VR SBIT + Relaxation	6 situations: Accommodation zone, food area with a virtual balance, room with posters, room with mirrors, a doorframe with strips and a zone to contrast body areas.	SBIT: 8 (3 h, weekly, group) Relaxation: 6 (1 h, weekly) VR: 6 (1 h, weekly).	-	BDI, PANAS, EAT, RS, BITE, EDI-2, BSQ, BIAQ, BAT, BES, BIATQ, ASI, SIBID, BASS.	VR condition participants showed a greater improvement in specific BI variables, depression and anxiety when compared to non-VR group. VR was a helpful tool for confronting the patients with BI distortions.
Riva et al. (2000) [34]	43 (0/43) Obese: 18 (0/18) BED: 25 (0/25)	Obesity (BMI>35) BED (DSM-IV)	NCCT	VEBIM 2 (ECT)	VEBIM 2 Different zones (Sitting-room, dining-room, kitchen, bedroom, working environments etc.).	5 (biweekly).	-	MMPI-2, EDI-2, BSS, BIAQ, FRS, CDRS.	In both groups, results showed a reduced level of body dissatisfaction, an improvement on social activities and a reduced use of disguising clothes. Obtained data were promising, but controlled and follow-up studies are needed to test this VR approach.
Riva et al. (2000) [35]	57 (0/57) Obese: 18 (0/18) BED: 25 (0/25) EDNOS: 14 (0/14)	Obesity (BMI>35) BED, EDNOS (DSM-IV)	NCCT	VEBIM 2	VEBIM 2 Different zones (Sitting-room, dining-room, kitchen, bedroom, working environments etc.).	5 (biweekly).	-	BSS, BIAQ, FRS, CDRS.	All groups showed a reduced level of body dissatisfaction, an improvement on social activities and a reduced use of disguising clothes. In the EDNOS group the reduction in body dissatisfaction was slighter than in other samples. VR-based treatment could be useful to BI modification in obesity, BED and EDNOS; but more studies are needed.
Riva et al. (2002) [36]	20 (0/20)	BED (DSM-IV)	RCCT	VR (+LCD+PT) PN (+LCD +PT)	VREDIM Different zones (Room with balance, drawing room, kitchen, dining room, bathroom, office, beach, supermarket etc.).	VR: 7 (50 min.) LCD: daily PT: NR (at least 30 min. walk/twice a week) PN: NR (3 times a week).	-	DIET, STAI, AI, WELSQ, URICA, BSS, BIAQ, FRS, CDRS.	VR treatment showed reduced level of body dissatisfaction and anxiety, increased self-efficacy and motivation for change, reduced concern about social judgment and reduced overeating. PN group presented anxiety reduction on the AI, but it was not confirmed by the STAI score. VR was more effective than PN on body satisfaction improvement, overeating and anxiety level reduction. VR-based therapy improved BI treatment.
Riva et al. (2003) [37]	36 (0/36)	BED (DSM-IV)	RCCT	ECT (+NG+PT) CBT (+NG +PT) NG (+PT) WL	VREDIM Different zones (Room with balance, drawing room, kitchen, dining room, bathroom, office, beach, supermarket etc.).	ECT: 15 (over 6 weeks) 10 VR sessions CBT: 15 (over 6 weeks) NG: 5 (weekly).	6 months	DIET, STAI, BDI II, RAS, RSEQ, WELSQ, URICA, BSS, BIAQ, CDRS.	ECT decreased anxiety and depression and improved assertive behavior. CBT decreased depression. NG decreased anxiety. All interventions groups improved patients’ self-esteem, eating control, eating self-efficacy, weight loss. They also reduced binge episodes. ECT was more effective than CBT on improving overall psychological state, body image, body satisfaction and resistance to social pressure. At follow-up, ECT improved body satisfaction and self-esteem and reduced the frequency of binge episodes when compared to CBT and NG. VR-based therapy improved BED patients’ treatment.
Riva et al. (2004) [38]	120 (0/120) Obese: 68 (0/68) BED: 36 (0/36) BN: 12 (0/12) EDNOS: 3 (0/3)	Obesity, BED, BN, EDNOS	RCCT	ECT (+NG+PT) CBT (+NG+PT) NG (+PT+PI) WL	VREDIM Different zones (Room with balance, drawing room, kitchen, dining room, bathroom, office, beach, supermarket etc.).	ECT: 15 (10 VR sessions of 15 min.) CBT: 15 NG: 4–6 PI: NR.	-	STAI, BDI, RSEQ, RAS, DIET, WELSQ, URICA, BSS, BIAQ, CDRS.	**ED:** ECT was the best intervention for eating control improvement. ECT and CBT were better on improving body satisfaction and body perception. ECT was better than CBT on BI and self-efficacy improvement. **Obesity:** All intervention groups helped on improving body satisfaction and body perception. ECT was the best intervention for eating control improvement. ECT and CBT were better to improve motivation to change. ECT was better than CBT on BI improvement. ECT was more effective than the others approaches.
Perpiñá et al. (2004) [42]	12 (0/12) AN: 7 (0/7) BN: 5 (0/5)	AN, BN (DSM-IV)	RCCT	SBIT+VR SBIT + Relaxation + VR	6 situations: Accommodation zone, food area with a virtual balance, room with posters, room with mirrors, a doorframe with strips and a zone to contrast body areas.	SBIT: 8 (3 h, weekly, group) Relaxation: 6 (1 h, weekly) VR: 6 (1 h, weekly).	6 months, 1 year	BIATQ, SIBID, ASI, BAT, EDI-2, EAT, BSI.	Improvement in all measures. Post-treatment results were maintained at follow-up, and for some measures like appearance-related schemas and ED related components the improvement continued along the follow-up period. General psychopathology improved from pre-treatment to one-year follow-up, and BSI score was always below the pre-treatment level, but it rose between 6 and 12 months. VR was useful in the treatment of BI and capable of enhancing the efficacy of the standard CBT.
Marco et al. (2013) [39]	18 (0/18)	BN, AN, EDNOS (DSM-IV-TR)	RCCT	CBT CBT (BI)+VR	5 situations: Virtual scale and kitchen, room with posters, room with mirrors, a doorframe with strips and a zone to contrast body areas.	CBT for BN:19 (Group, 2 h, weekly) CBTC for AN:23 (individual) CBT: 15 (group) + VR: 8 (1 h, weekly).	1 year	BAT, BIATQ, BASS, SIBID, BITE, EAT.	CBT+VR showed more BI improvement than CBT; CBT+VR showed more body attitudes and frequency of negative automatic thoughts on BI improvement at post-treatment and this continued to rise at follow-up; more body satisfaction, discomfort caused by body-related situations and BN symptoms (BITE) improvement at post-treatment and follow-up; greater improvement in the behavior clinical measures. All participants improved in the ED measures and it was maintained at follow-up. CBT+VR post-treatment and follow-up showed clinically significant improvement and all BI and ED scores were similar to healthy population. CBT+VR also showed EAT better scores than healthy population. BITE normalization was achieved at follow-up. CBT focused on BI plus VR improved CBT standard treatment for ED.
Cesa et al. (2013) [40]	66 (0/66)	Obesity + BED (DSM-IV-TR)	RCCT	ECT (+IP) CBT (+IP) IP	Neuro-VR 14 environments (Home, Supermarket, Pub, Restaurant, Swimming Pool, Beach, Gymnasium, BI comparison areas).	ECT: 15 (5 weeks, 10 biweekly VR sessions) CBT:15 (5 weeks) IP (6 weeks).	1 year	EDI-Symptom Checklist, BSS, BIAQ, CDRS.	Weight decreased, number of binge eating episodes decreased to zero, body satisfaction improved in all groups. BI concerns improved only in ECT. ECT and CBT were more effective than IP alone in preventing weight regain at follow-up. Only ECT was effective in further weight loss. Binge eating episodes were reported at follow-up, ECT and CBT were successful in maintaining them at a low rate.
Roncero and Perpiñá (2015) [41]	1 (0/1)	BN (DSM-IV-TR)	CR	CBT+VR	Kitchen with two areas that included elements to cook, drink and eat.	CBT:7 VR:7 (60 min/weekly or biweekly).	-	EDI-2, BITE, ACTA, BDI-2, BAI.	Reduction in binges, purges and food avoidance; development of the ability to make decisions over impulses.

**Note: Participants**—AN: Anorexia Nervosa; BED: Binge Eating Disorder; BN: Bulimia Nervosa; ED: Eating disorder; EDNOS eating disorder not otherwise specified; **Intervention**—CBT: Cognitive Behavior Therapy; ECT: Experiential Cognitive Therapy; IP: Integrated multimodal medically managed inpatient program; LCD: Low-calorie Diet; PI: Psychological support; PN: Psycho-nutritional intervention; SBIT: standard BI treatment; VEBIM: Virtual Reality for Body Image Modification; VREDIM: Virtual Reality for Eating Disorders Modification; WL: Waiting List Group; **Study Design**—CR: Case Report; NCCT: Non-Controlled Clinical Trial; RCCT: Randomized Controlled Clinical Trial; **Instruments**—ACTA: Attitudes Toward Chance in eating disorders; AI: Assertion Inventory; ASI: Appearance Schemas Inventory; BAI: Beck Anxiety Inventory; BAT: Body Attitudes Test; BASS: Body Areas Satisfaction Scale; BDI II: Beck Depression Inventory; BIAQ: Body Image Avoidance Questionnaire; BIATQ: Body Image Automatic Thoughts Questionnaire; BITE: Bulimic Investigatory Test; BSI: The Brief Symptom Inventory; BSS: Body Satisfaction Scale; CDRS: Contour drawing rating scale; DIET: Dieter’s Inventory of Eating Temptations; EAT: Eating Attitudes Test; EDI 2: Eating Disorders Inventory 2; EDI-Symptom Checklist: Eating Disorder Inventory-Symptom Checklist; FRS: Figure rating scale; MMPI 2: Minnesota Multiphasic Personality Inventory 2; PANAS: Positive and Negative Affect Schedule; RAS: Rathus Assertiveness Schedule; RSEQ: Rosenberg Self-Esteem Questionnaire; SIBID: Situational Inventory of Body Image Dysphoria; STAI: Sate-Trait Anxiety Inventory; URICA: University of Rhode Island Change Assessment Scale; WELSQ: Weight Efficacy Life-Style Questionnaire; **Other**—BMI: Body Mass Index; BI: Body Image; CG: Control Group; ClG: Clinical Group; F: Female; M: Male; NG: Nutritional Group; PT: Physical Training; SCID-I: Structured Clinical Interview for DSM-IV Axis I Disorders.
